# Gamma glutamyl transferases in association with cardiovascular risk scores in non-diabetic hypertensive Cameroonians: preliminary data from HYRICCA study

**DOI:** 10.1186/s13104-022-06190-1

**Published:** 2022-09-15

**Authors:** Jan René Nkeck, Chemgne Marie Ida, Valerie Ndobo Koe, Antonin Wilson Ndjitoyap Ndam, Yondo Ndedi Claudine Jessica, Eko Ondoa Manuella, Boukeu Yonta Charelle, Zouague Zalbi Corine, Ntyam Abena Andrée, Falmata Amazia, Jériel Pascal Nkeck, Esther Astrid Mbono Samba, Vicky Jocelyne Ama Moor

**Affiliations:** 1 Faculty of Medicine and Biomedical Sciences, University of Yaoundé 1, Yaoundé, Cameroon; 2 Groupe de Recherche HYRICCA (Hypertension et Risque Cardiovasculaire des Camerounais), Yaoundé, Cameroon; 3 Cardiology Department, Yaoundé Central Hospital, Yaoundé, Cameroon; 4 Biochemistry Laboratory of the Yaoundé University Hospital Centre, Yaoundé, Cameroon

**Keywords:** Gamma glutamyl transferase, Hypertension, Cardiovascular risk, Cameroonians

## Abstract

**Objective:**

The usefulness of gamma glutamyl transferase (GGT) as biomarker of cardiovascular risk (CVR) remains unexplored in sub-Saharan Africans. To evaluate their relevance on CVR assessment in non-diabetic hypertensive Cameroonians. This was a prospective cross-sectional study on non-diabetic hypertensive adults aged 57.7 ± 10 years (62% female), without evidence of acute or chronic liver disease, in which we assessed GGT levels and correlates it with validated CVR biomarkers, CVR scores (WHO risk score, Framingham 2008, ASCVD 2013, EuroSCORE 2003, and Reynolds score), and plasma atherogenic index (PAI).

**Results:**

We found a positive but weak association between GGT and PAI on linear regression [0.004 (0.001; 0.007); p = 0.021], which was dependent of triglycerides levels (r = 0.17; p = 0.03). We did not find a significant association between GGT levels and the results of the CVR scores studied; Although being related to atherogenic risk, as reported in literature in non-sub-Saharan Africans, GGTs would be of little value for CVR assessment in our population.

## Background

The epidemiological and socioeconomic burden of hypertension is constantly increasing throughout the world, particularly in Sub-Saharan Africa (SSA), where a large majority of hypertensive patients are unaware of their condition, the remaining proportion being mostly poorly controlled, all of them being exposed to a significant risk of premature death [1]. In Cameroon in 2019, Dzudie et al. found a prevalence of 20.8% in the population, i.e. almost one adult in four [2]. Hypertension must be prevented, detected and treated, but this does not limit itself to the blood pressure figure, the overall cardiovascular risk (CVR) must be reduced, which is a major concern for the physician nowadays. The assessment of CVR involves the screening for cardiovascular risk factors (CRFs), the measurement of CVR markers, and the integration of these into a standardized cardiovascular risk equation in order to make a consensus treatment decision [3]. However, there is not yet a validated equation in all the sub-Saharan groups and the measurement of many CVR markers is costly in practice. It therefore seemed important to evaluate the usefulness of certain non-conventional markers that can be routinely performed in this context, such as gamma glutamyl transferases (GGT).

The evaluation of GGT activity is mainly performed in the context of the diagnosis of liver and biliary tract diseases. However, several authors have emphasized its involvement in the pathogenesis of cardiovascular disease (CVD), in particular its antioxidant role in maintaining glutathione homeostasis by ensuring the assimilation of amino acids and their reuse in its intracellular synthesis [4]; and its potential role in the initiation and progression of atherosclerosis, either through the production of free radicals or through a modification of LDL (low-density lipoprotein) cholesterol [5]. Increased GGT activity may be associated with an increase in CVR in hypertensive patients, independent of age, sex and alcohol consumption [6]. However, in our context of high prevalence of viral hepatitis and high alcohol consumption, it is important to analyse the potential contribution of its evaluation in order to guide the clinician on its potential usefulness in the CVR of hypertensive patients. The aim of the present study is therefore to evaluate the relevance of GGT measurement in the assessment of cardiovascular risk in nondiabetic hypertensive Cameroonians.

## Methods

### Study design and setting

We carried out a prospective cross-sectional study in the Cardiology Department of the Yaoundé Central Hospital, and the Biochemistry Laboratory of the Yaoundé University Hospital Centre. Study participants were recruited from January 2022 to March 2022. This study was conducted within the unfunded project HYRICCA (*Hypertension et Risque Cardiovasculaire des Camerounais*).

### Participants

We included consenting adults aged above 18 years living with hypertension diagnosed according to the ESC/ESH (European Society of Cardiology/European Society of Hypertension) 2018 criteria [7]. We excluded any participant with known liver disease (including viral hepatitis B and C) or clinical signs of it, and/or alanine amino transferase (ALT) levels greater than 1.5 times the upper limit of normal (36U/L). We also excluded any participant with known diabetes, or with a plasma fasting glucose level greater than 1.26 g/L, chronic kidney disease (CKD) with glomerular filtration rate less than 45 mL/min (evaluate with the MDRD (Modification of Diet in Renal Disease) formula with 4 parameters), as well as any participant on anticonvulsant medication, hormonal contraception, thyroid hormones, non-steroidal anti-inflammatory medication, and/or having had an infection in the previous month, or suffering from a chronic inflammatory disease or cancer, as these situations can altered the GGT and inflammatory markers levels.

### Sample size estimation

The sample size was estimated at 146 using the sample size formula for a difference in means contained in Whitley and Ball's article [8]. The standardized difference was calculated from the GGT values contained in the Melvin et al. study between the higher and lower cardiometabolic risk groups (included hypertensive), for 80% power and a 0.05 error rate [9].

### Data collection

Participants were identified during their cardiology consultation and invited to participate in the study. An informed consent was obtained from each participant before inclusion. Data were collected using a data collection sheet. These included sociodemographic (age, gender) and clinical data. Clinical variables includes history of hypertension (time since diagnosis, current treatment), other cardiovascular risk factors (smoking, alcohol consumption, sedentary lifestyle, known dyslipidaemia, first-degree family history of major cardiovascular event), cardiovascular complications, comorbidities, blood pressure (BP), weight and height to calculate the body mass index (BMI), and the waist circumference (WC). Blood pressure was measured using the device OMRON^®^ M7 Brand digital blood pressure monitor, INC.

### Biological analysis

After the clinical examination, we collected 15 mL of fasting plasma blood (8 h fasting). On this sample, we performed GGT assays according to the method of Szasz, Rosalki, and Tarlow [10]; fasting blood glucose according to the method of Trinder; albuminemia according to the Bromocresol Green’s method (BCG); serum uric acid according to the Uricase method; serum creatinine by the modified Jaffé kinetic and colorimetric method (used to calculate glomerular filtration rate later using the 4-parameter MDRD equation); high-sensitive C-reactive protein (hsCRP) by immunofluorescence, and lipid profile components (total cholesterol, triglycerides, and HDL cholesterol) according to the method of Trinder. LDL cholesterol was calculated using the Friedewald formula. From the lipid profile parameters, we calculated the atherogenicity indices: ratio of total cholesterol to HDL, LDL to HDL ratio, triglycerides to HDL ratio, and the plasma atherogenic index (PAI) (logarithm of triglycerides to HDL ratio).

### Evaluation of cardiovascular risk

For each participant, we assessed global cardiovascular risk using five model scores, namely the WHO (World Health Organization) CVD risk laboratory-based charts for central Sub Saharan Africa (for participants aged 40 to 74 years), the EuroScore 2003 (for men aged 40 to 65 years and women aged 50 to 65 years), the ACC/AHA ASCVD (American College of Cardiology/American Heart Association Atherosclerotic Cardiovascular Disease) score 2013 (patients over 40 years of age), the Framingham score 2008 (30 to 79 years of age), and the Reynolds score (for participants under 80 years of age) [11–14]. These scores were selected from the literature with a preference for those that included black subjects in their study population. From these scores, each participant was classified according to his or her overall cardiovascular risk and stratified according to their recommendations.

### Operational terms

Sedentary lifestyle was defined as physical activity of less than 45 min twice a week. Abdominal obesity was considered for a waist circumference exceeding 102 cm in men and 88 cm in women; overweight was defined as a BMI between 25 and 29.9 kg/m^2^, and obesity as a BMI of 30 kg/m^2^ or more. Controlled hypertension was defined for systolic and diastolic values below 140 and 90 mmHg, respectively for a hypertensive patient. Moderate fasting hyperglycaemia was considered for fasting blood glucose between 1.1 and 1.25 g/L. Hyperuricemia was considered above 60 mg/L for women and 70 mg/L for men. Hypercholesterolemia above 2 g/L total cholesterol; low HDL cholesterol below 0.45 g/L in men and 0.55 g/L in women; hypertriglyceridemia above 1.6 g/L in men and 1.35 g/L in women; and hyper LDL cholesterol above 1.3 g/L. HsCRP values exceeding 6 mg/L were excluded. Metabolic syndrome was defined according to the NCEP-ATP III criteria [15].

### Statistical analysis

All the data collected were analysed using the software SPSS version 23.0. The figures have been designed with Microsoft^®^ Office Excel software version 2016. Quantitative variables are presented with their mean and standard deviation (SD), or median with interquartile range for non-continuous variables. Qualitative variables were expressed as counts and proportions. Association with GGT and various clinical and biological parameters of cardiovascular risk, were sought using the Pearson’s correlation coefficient (r). We used linear regression to analyse the effect of GGTs on the different cardiovascular risk scores through three models: an unadjusted model, and two models adjusted on the one hand by age and gender, and on the other hand by age, gender, uric acid, HDL, LDL, triglycerides, ALT, hsCRP, and waist circumference. Furthermore, using the Anova test, we compared the risk levels according to the studied scores based on the GGT values stratified according to the interquartile ranges. For all the tests used, the threshold of significance was set at 0.05.

## Results

### Characteristics of the sample

Of 180 participants included for the study, 163 were finally retained. The sample includes adults aged 57.7 (10.4) years on average (min–max: 29–84 years), with 101 (62%) women, and a median duration of hypertension of 7 [3; 15] years. Nine out of ten participants were on pharmacologic treatment for hypertension and 41 (25.2%) were controlled during the study. Most of them received dual therapy (72; 44.2%) or monotherapy (48; 29.4%); and mostly comprising a calcium channel blocker (92; 62.2%), a diuretic (78; 52.7%) and/or a renin angiotensin aldosterone system blocker (66; 44.6%). The most frequent CVRFs were sedentary lifestyle (73; 44.8%) and family history of a major cardiovascular event at the first-degree (43; 29.4%). Sixty-one (37.4%) participants had a metabolic syndrome, and 25 (15.3%) participants had already had a cardiovascular event, mainly stroke (12; 7.4%) and hypertensive retinopathy (10; 6.1%). The most common comorbidities were osteoarthritis (18; 11%) and gout (5; 3.1%). Overweight and obesity affected 44.8% and 39.3% of participants respectively, with 65.6% abdominal obesity. Moderate fasting hyperglycaemia was found in 7 (4.3%) participants and we observed 41 (25.2%) cases of hyperuricemia, and several cases of dyslipidaemia including low HDL cholesterol (97; 59.3%), elevated LDL cholesterol (48; 29.4%), and hypercholesterolemia (43; 26.4%). The principal characteristics of the sample are summarised in Table 1.

**Table 1:** Clinical and biological characteristics of participants

Variables	N (%) or Mean (SD)
N (%)	163 (100)
Female gender, n (%)	101 (62)
Age, Mean (SD), years	57.7 (10.4)
HTN medication, n (%)	148 (90.8)
Tobacco consumption, n (%)	3 (1.8)
Alcohol consumption, n (%)	37 (22.7)
Sedentary lifestyle, n (%)	73 (44.8)
Known dyslipidemia, n (%)	7 (4.3)
Familial history of CV events, n (%)	43 (26.4)
Vascular complications, n (%)	25 (15.3)
Comorbidities, n (%)	40 (24.5)
Systolic BP (SD), mmHg	150 (21.9)
Diastolic BP (SD), mmHg	95.2 (13.1)
BMI (SD), Kg/m^2^	29.2 (4.8)
WC (SD), cm	100.2 (11.6)
Metabolic syndrome	61 (37.4)
Glycaemia (SD), g/L	0.86 (0.13)
HDL cholesterol (SD), g/L	0.49 (0.14)
LDL cholesterol (SD), g/L	1.12 (0.45)
Total Cholesterol (SD), g/L	1.79 (0.44)
Triglycerides (SD), g/L	0.82 (0.40)
GGT (SD), U/L	29.3 (11.7)
ALT (SD), U/L	17 (9.3)
Serum albumin (SD), g/L	42.6 (2.6)
Uricemia (SD), mg/L	54.7 (16.3)
hsCRP (SD), mg/L	1.6 (1.29)

*Min* minimum, *Max* maximum, *SD* Standard deviation, *IQR* Interquartile range, *HTN* hypertension, *CV* *event* cardiovascular event, *BP* blood pressure, *BMI* body mass index, *WC* waist circumference, *HDL* high-density lipoprotein, *LDL* low-density lipoprotein, *GGT* gamma glutamyl transferase, *ALT* alanine amino transferase, *hsCRP* high sensitive C-reactive protein

### Association of GGT with CVR markers and CVR scores

We first analyzed the correlation between GGT values and clinical and biological markers of cardiovascular risk assessed during the study. We found a positive and weak correlation with triglyceride levels (r = 0.17; p = 0.03), triglyceride to HDL ratio (r = 0.202; p = 0.01), and the PAI (r = 0.18; p = 0.02) (Table 2). Subsequently, we compared the cardiovascular risk of the participants according to the GGT values subdivided into interquartile ranges (Table 3). We found that there was no difference in overall cardiovascular risk between the different groups of interquartile ranges. Finally, we studied the impact of GGT variations on overall cardiovascular risk according to the three linear regression models presented in the methodology (Table 4). We observed a significant association between GGT levels and the atherogenic plasma index [0.004 (0.001; 0.007); p = 0.021] independently with sex and age, but dependent on the triglyceride level with which GGT was significantly correlated as seen in Table 2.

**Table 2:** Evaluation of association with gamma glutamyl transferases and various clinical and biological parameters of cardiovascular risk

Variables	GGT	
	Correlation coefficient (r)	p-value
Age	− 0.053	0.49
HTN duration	− 0.070	0.37
Systolic BP	0.076	0.33
Diastolic BP	0.066	0.40
WC	0.084	0.28
BMI	0.092	0.24
Glycaemia	0.125	0.11
Uricemia	0.145	0.06
HDL cholesterol	− 0.128	0.10
Triglycerides	**0.170**	**0.03**
Total cholesterol	− 0.092	0.241
LDL cholesterol	− 0.059	0.45
TC to HDL ratio	0.033	0.67
LDL to HDL ratio	0.009	0.907
TG to HDL ratio	**0.202**	**0.01**
PAI	**0.181**	**0.02**
hsCRP	0.039	0.66

*GGT* gamma glutamyl transferase, *HTN* hypertension, *BP* blood pressure, *WC* waist circumference, *HDL* high-density lipoprotein, *LDL* low-density lipoprotein, *TC* total cholesterol, *TG* triglycerides, *hsCRP* high sensitive C-reactive protein

**Table 3:** Comparison of various cardiovascular risk according to the stratified GGT levels

Variables	GGT, mean (SD) (UI/L)				
	N	< Q25	[Q25–Q75]	≥ Q75	p-value
WHO risk score	148	11 (6)	11 (6)	11 (6)	0.94
EuroScore 2003	117	3.6 (3.2)	3.6 (2.9)	2.6 (2.6)	0.42
Framingham score 2008	162	15.3 (8.5)	15.7 (8.5)	15.9 (8.3)	0.94
ASCVD score 2013	150	12.5 (7.5)	14.4 (13)	14 (8.2)	0.69
Reynolds score	138	10 (18.6)	7.1 (7.2)	9.4 (12.7)	0.44
Plasma atherogenic index	163	0.13 (0.24)	0.22 (0.26)	0.24 (0.25)	0.09

**Table 4:** Results of linear regression analysis of GGT levels and various cardiovascular risk scores

Variables	Model 1	Model 2	Model 3
Framingham score (2008)	0.64 (− 0.06; 1.56) p = 0.42	0.075 (− 0.013; 0.16) p = 0.09	0.035 (− 0.048; 0.11) p = 0.4
ASCVD/AHA (2013)	0.048 (− 0.097; 0.19) p = 0.51	0.1 (− 0.025; 0.23) p = 0.11	0.067 (− 0.06; 0.19) p = 0.32
Reynolds score	0.006 (− 0.17; 0.19) p = 0.84	0.016 (− 0.16; 0.19) p = 0.17	− 0.038 (− 0.22; 0.14) p = 0.68
WHO risk score	− 0.007 (− 0.089; 0.076) p = 0.86	0.042 (− 0.013; 0.09) p = 0.13	0.03 (− 0.029; 0.089) p = 0.31
EuroScore 2003	− 0.016 (− 0.06; 0.028) p = 0.47	0.009 (− 0.027; 0.045) p = 0.64	0.004 (− 0.035; 0.042) p = 0.85
Plasma atherogenic index	**0.004 (0.001; 0.007) ** **p = 0.021**	**0.004 (0.001; 0.007) ** **p = 0.017**	0.001 (− 0.001; 0.001) p = 0.37

Model 1: unadjusted; Model 2: adjuster for age and gender; Model 3: Model 2 + adjustment for HDL, LDL, triglycerides, ALT, WC, hsCRP; Non standardized beta coefficient and its 95% confidence interval

## Discussion

Cardiovascular risk assessment of hypertensive patients is a concern for the physician, especially in the context of limited resources where there is a need to continuously improve cost-effective strategies to achieve optimal assessment. The present study examined the value of GGT in this regard in a sub-Saharan African population. We found that GGTs have a slight association with atherogenic indices dependent on triglycerides, but not with the overall cardiovascular risk. We will discuss our results by focusing on the links sought with conventional biomarkers and CVR scores.

Chronic low-grade inflammation makes a strong contribution to the phenomenon of atherosclerosis and CVR. One of the indicators of this phenomenon is the ultrasensitive CRP, which studies have already proven to be a biomarker of choice in this sense. Several studies in the literature have raised the link between GGT and chronic subclinical inflammation making GGT especially in the case of metabolic syndrome in asymptomatic populations and type 2 diabetics [9, 16, 17]. However, we did not find in our sample a significant correlation between GGT and hsCRP. The association between chronic inflammation and GGT would probably be more through insulin resistance and metabolic syndrome, but having worked on a population of non-diabetic hypertensives and having few pre-diabetic states in our sample this could explain this difference [18]. Furthermore, GGT would be more of a marker of cardiometabolic risk in the literature, but we had few major cardiovascular events in our sample [19]. In sum, the association between GGT and ultrasensitive CRP remains complex and not fully understood and there is a need for more studies to better understand these interactions [20].

The pathogenesis of atherosclerosis is related to cholesterol metabolism and oxidative stress. Being an antioxidant, we evaluated the association between serum GGT levels and markers of lipid profile and found a positive but weak association with triglyceride levels, triglyceride to HDL ratio and atherogenic plasma index. These data are consistent with those already present in the literature, which found a significant and positive association between GGT and lipid profile parameters, as well as atherogenicity indices [21, 22]. The elevation of GGT is considered as a risk factor for atherogenicity, and this is better understood when its effect on oxidative stress is analysed. Indeed, glutathione will be degraded by GGT, which will eventually lead to the synthesis of free cysteine and glycine, which in the extracellular compartment can participate in peroxidation reactions with oxidation of LDL and contribute to the formation and instability of atherosclerotic plaque that can lead to coronary heart disease and other vascular events [18].

We assessed the value of GGT as a predictive marker of cardiovascular risk according to the cardiovascular risk assessment models in the literature. We did not find any association in this regard. Although a positive and weak correlation was observed using the GGT to HDL ratio, it is clear that this association is more the result of HDL cholesterol. The utility of GGT as a predictive marker of cardiovascular risk is not consensual in the literature. Several observational studies have demonstrated a positive and linear independent association with cardiovascular disease risk, although in the general population the use of GGT would not ultimately improve the prediction of cardiovascular risk [23–25]. Also, in some populations, GGTs have not been shown to be significantly associated with cardiovascular mortality, especially in Asians [24].

## Limitations

The interpretation of these data must, however, take into account certain limitations related to the fact that multiple GGT assays were not performed, since its values may vary and it is important to have several assays in order to ensure the baseline status. However, we have been very careful to eliminate many confounding factors that could hinder its assessment. In addition, the small sample size, and the assessment of global cardiovascular risk using risk equations present in the literature but never validated properly in our population. Nevertheless, we have chosen those that include at least blacks in their source population and the WHO risk score which was developed including population of Central Sub-Saharan Africa; we believe that, pending data from risk assessment models specific to our population, this already gives an overview of the overall cardiovascular risk.

Taking into consideration our results and their limitations, we can conclude that GGTs are slightly associated with atherogenic risk, but may not contribute to the overall cardiovascular risk of non-diabetic hypertensive Cameroonians. Further studies are needed for a better assessment of the influence of GGTs on the overall cardiovascular risk.

## Data Availability

The datasets used for this study are available from the corresponding author on reasonable request.

## Abbreviations

ACC: American college of cardiology
AHA: American heart association
ALT: Alanine amino transferase
ASCVD: Atherosclerosis cardiovascular diseases
BMI: Body mass index
BP: Blood pressure
CVD: Cardiovascular disease
CRF: Cardiovascular risk factor
CVR: Cardiovascular risk
ESC: European Society of Cardiology
ESH: European Society of Hypertension
GGT: Gamma glutamyl transferase
HDL: High-density lipoprotein
hsCRP: High sensitive C-reactive protein
HYRICCA: *Hypertension et Risque Cardiovasculaire des Camerounais*
LDL: Low-density lipoprotein
PAI: Plasma atherogenic index
SSA: Sub-Saharan Africa
WC: Waist circumference
WHO: World Health Organization
